# Supplementary material for: Iron disproportionation in peridotite fragments from the mantle transition zone
Source: Nat Commun. 2025 Jul 1;16:5440. doi: 10.1038/s41467-025-60566-y (PMC12215351; doi:10.1038/s41467-025-60566-y)
Supplement: Supplementary file 1 — Supplementary Information [file 41467_2025_60566_MOESM1_ESM.docx]

Supporting Information for

**Iron disproportionation in peridotite fragments from the mantle transition zone**

Fabin Pan^1^, Xiang Wu^1^[[1]](#footnote-1)^*^, Chao Wang^1^, Yanfei Zhang^1^, Yiping Yang^2^, Xiaobo He^3^, Chong Jin^4^, Lian Zhou^1^, Hongfei Zhang^1^, Hongping He^2^, Junfeng Zhang^1*^

^1^ *State Key Laboratory of Geological Processes and Mineral Resources, and School of Earth Science,* *China University of Geosciences, Wuhan 430074, China*

^2^ *State Key Laboratory of Deep Earth Processes and Resources,* *Guangzhou Institute of Geochemistry, Chinese Academy of Sciences, Guangzhou 510640, China*

^3^ *Marine Science and Technology College, Zhejiang Ocean University, Zhoushan 316022, China*

^4^ *Zhejiang Institute of Geosciences, Hangzhou 310007, China*

**Contents of this file**

Supplementary Figures 1 to 8

**Introduction**

This supporting information provides the Supplementary Figure 1 to 8.

Supplementary Figures 1 to 8


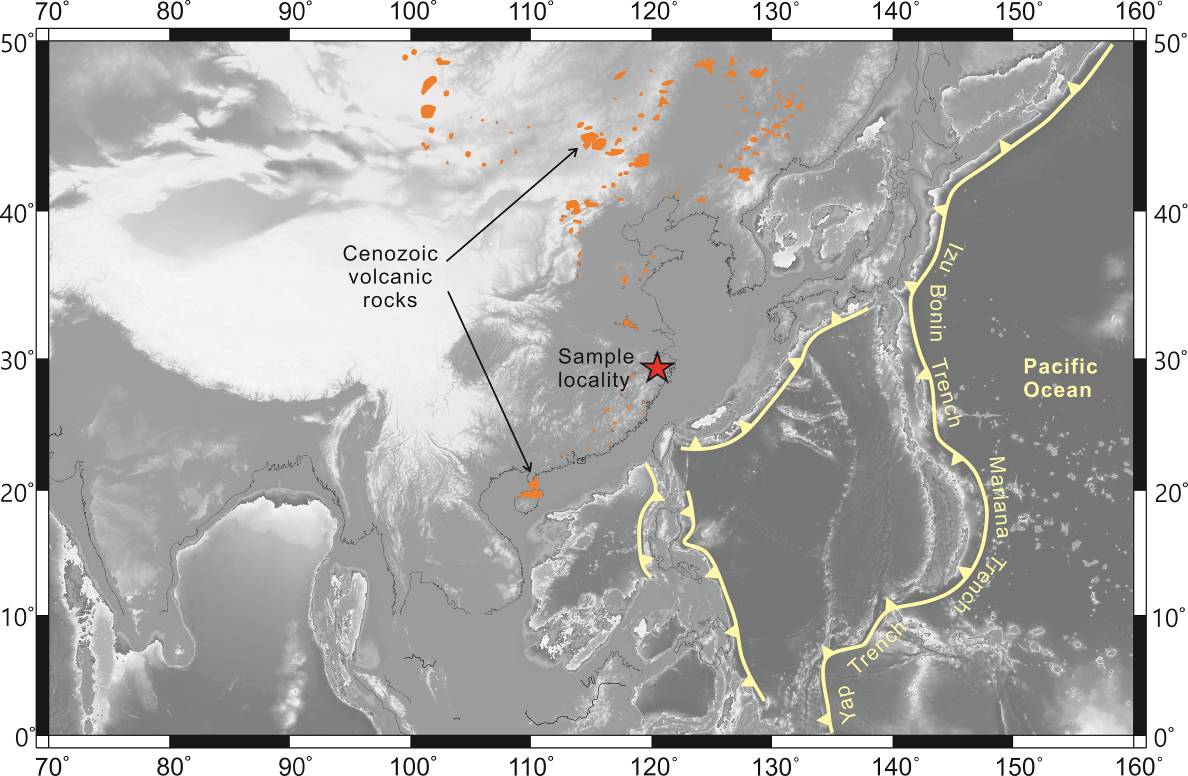


**Supplementary Figure 1.** **Topography map showing locations of the studied mantle xenolith samples (red star) and Cenozoic volcanic rocks (red patches) along eastern Asian continental margin.** The mantle xenoliths were collected from Cenozoic alkaline basalts in Zhejiang Province, Southeast China (Pan et al., 2021)^14^. Surface topography data are sourced from the Generic Mapping Tools (GMT)^58^.


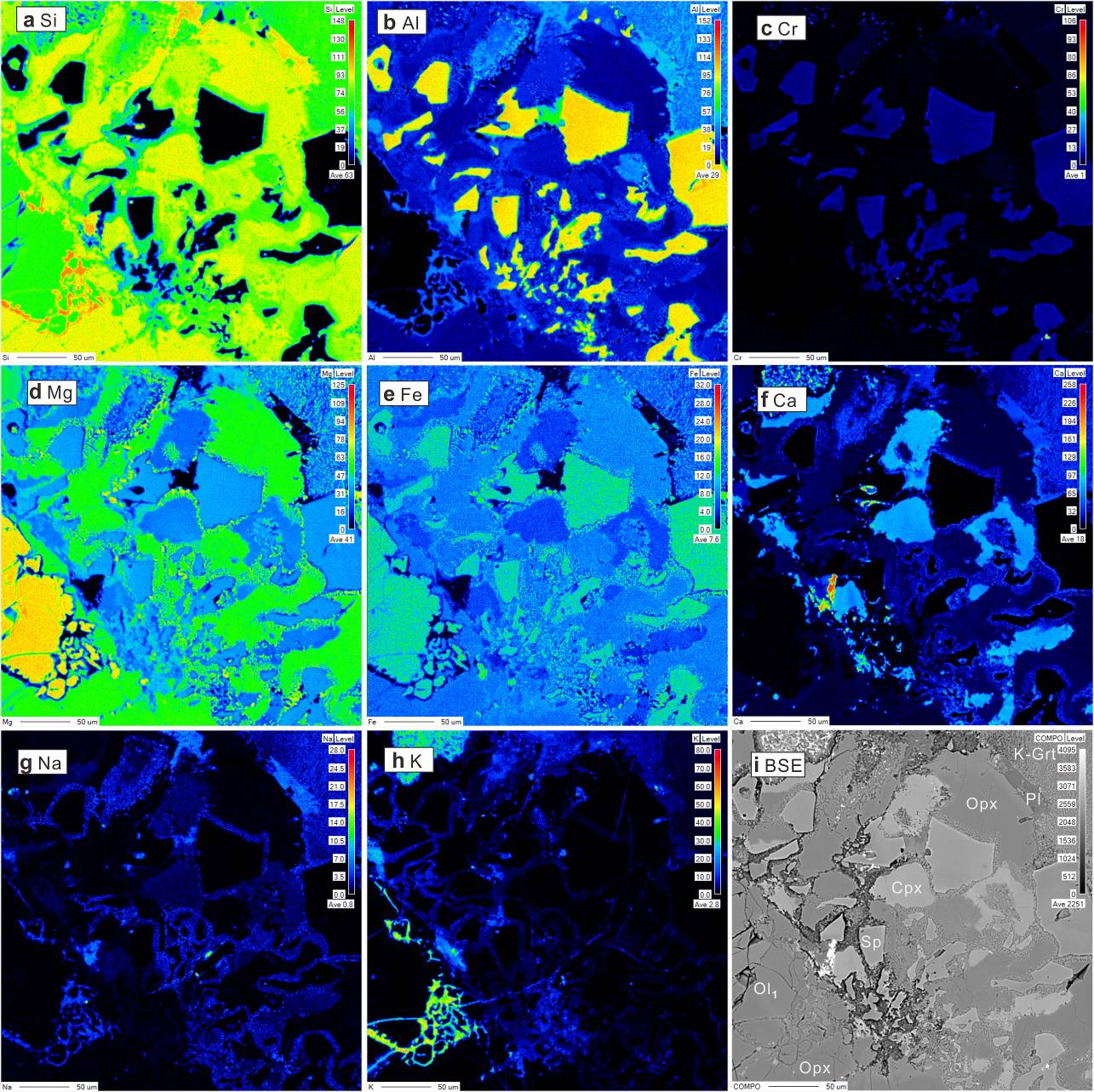


**Supplementary Figure 2.** **Elemental maps of a kelyphitized garnet rim obtained by electron microprobe analysis (EPMA).** (a) silicon (Si); (b) aluminum (Al); (c) chromium (Cr); (d) magnesium (Mg); (e) iron (Fe); (f) calcium (Ca); (g) sodium (Na); (h) potassium (K); (i) back-scatter electron (BSE) image. The K-Grt rim consists of orthopyroxene (Opx), clinopyroxene (cpx), spinel (Sp), and plagioclase (Pl).

**Supplementary Figure 3. Microstructural and elemental characteristics of a kelyphitized garnet core.** (a) Band contrast (BC) image; (b) phase map; (c) Euler image; (d-k) elemental maps. Note that the examined area encompasses a domain composed of Opx + Sp + Na-rich matrix on the left, and another domain on the right characterized by Ol + Sp + Na-rich matrix, featuring slightly larger grains.


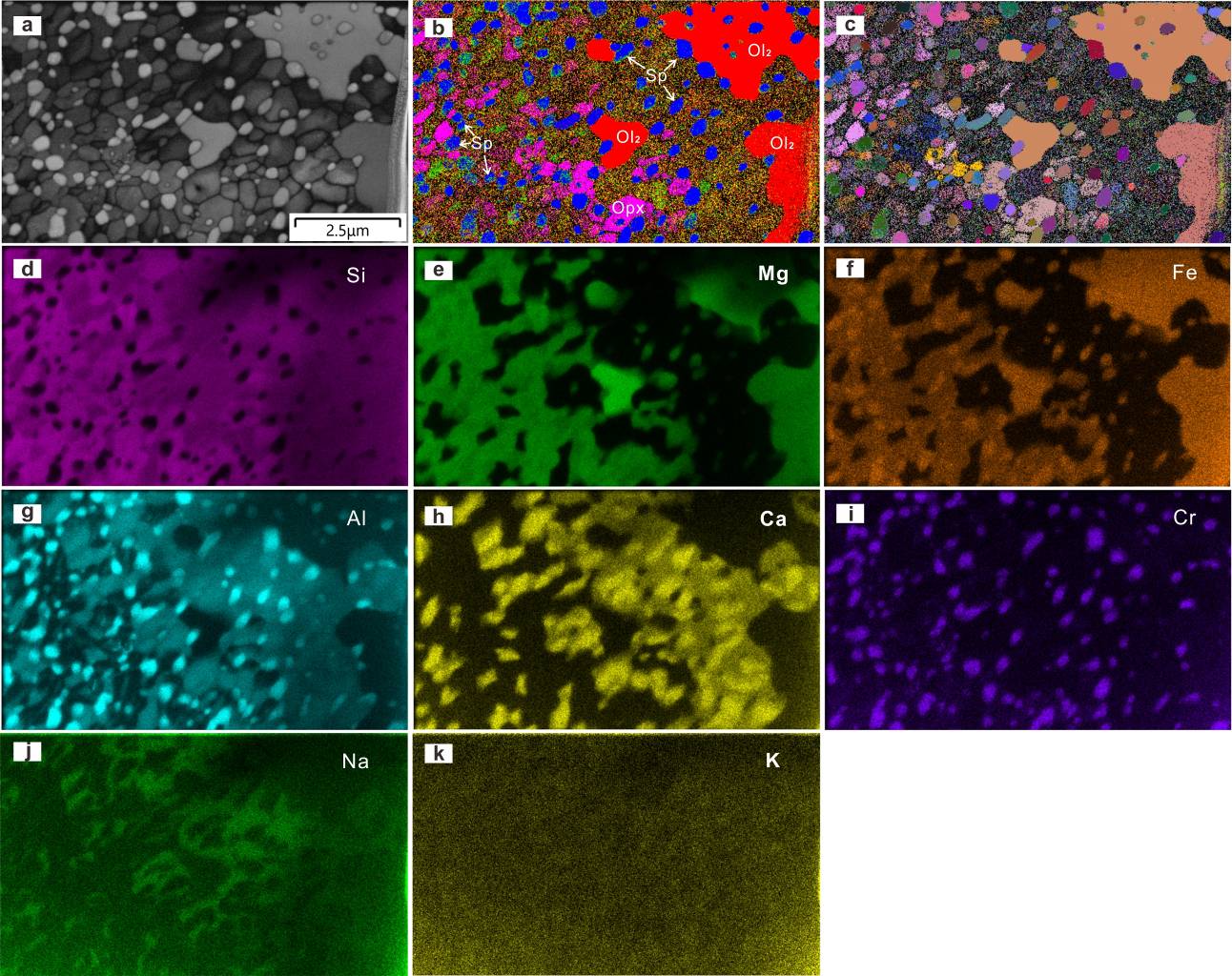

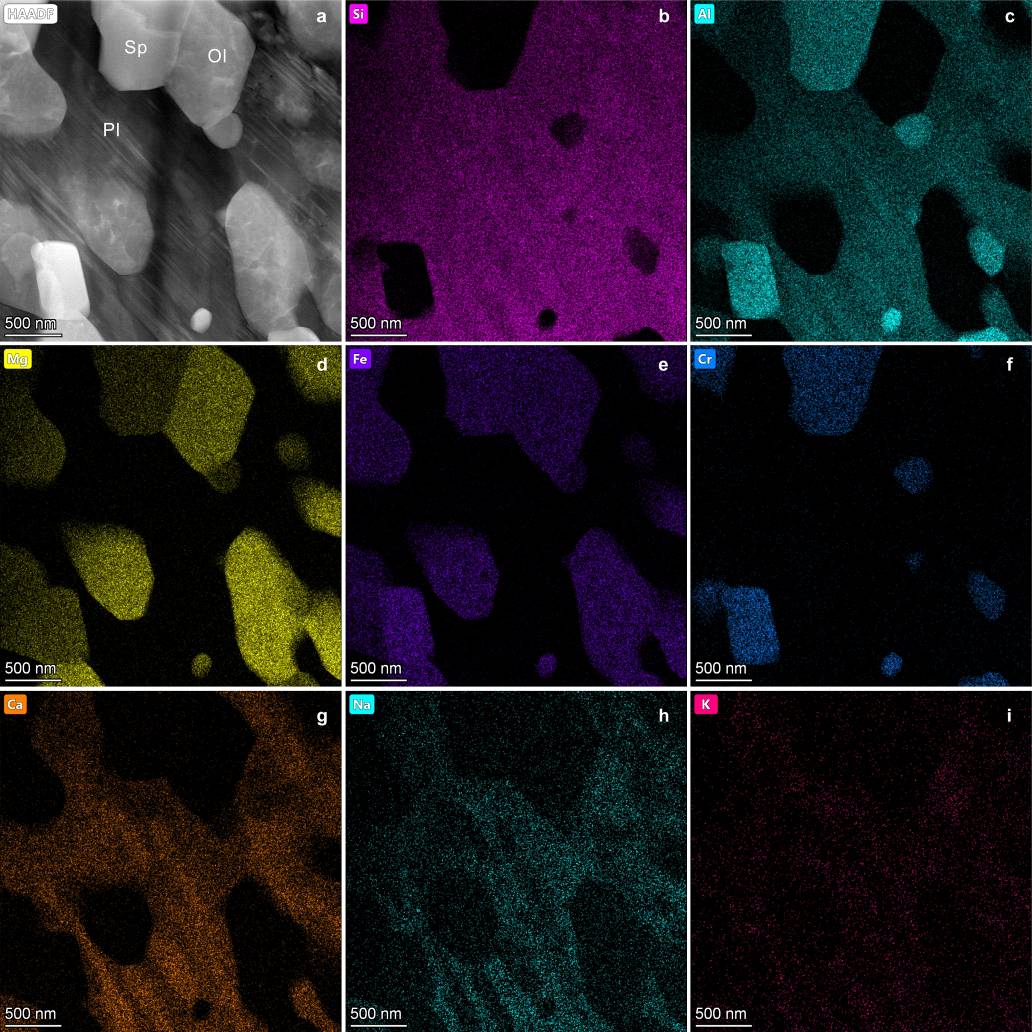


**Supplementary Figure 4. Transmission electron microscopy analyses of the kelyphitized garnet core.** Panel (a) is a HAADF-STEM micrograph, while panels (b-i) are EDS elemental maps.


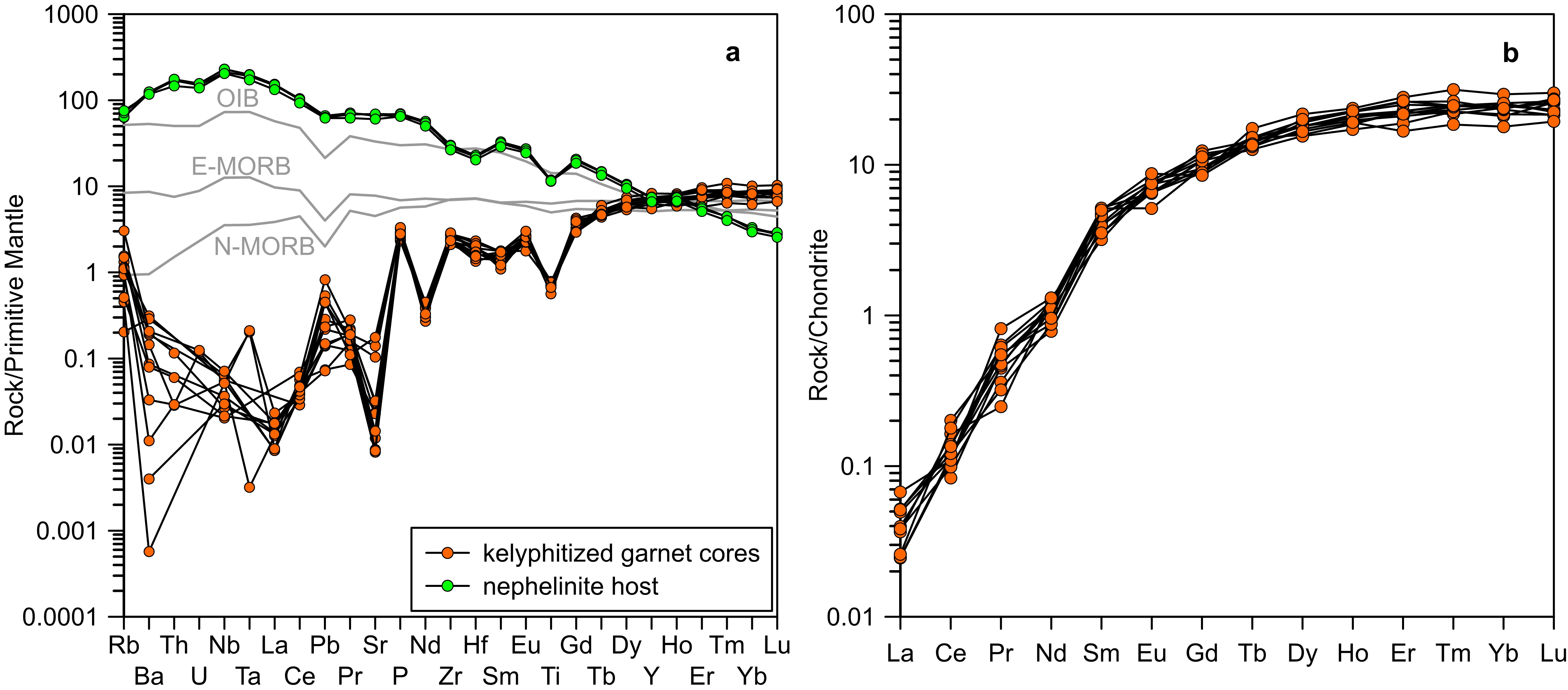


**Supplementary Figure 5. Trace element compositions of kelyphitized garnet cores and their nephelinite host.** (a) Spider diagrams of primitive mantle-normalized element concentrations; (b) Chondrite-normalized REE patterns. Normalizing values are sourced from Sun and McDonough (1989)^59^. OIB: oceanic island basalts; N-MORB: normal mid-ocean ridge basalts; E-MORB: enhanced mid-ocean ridge basalts.


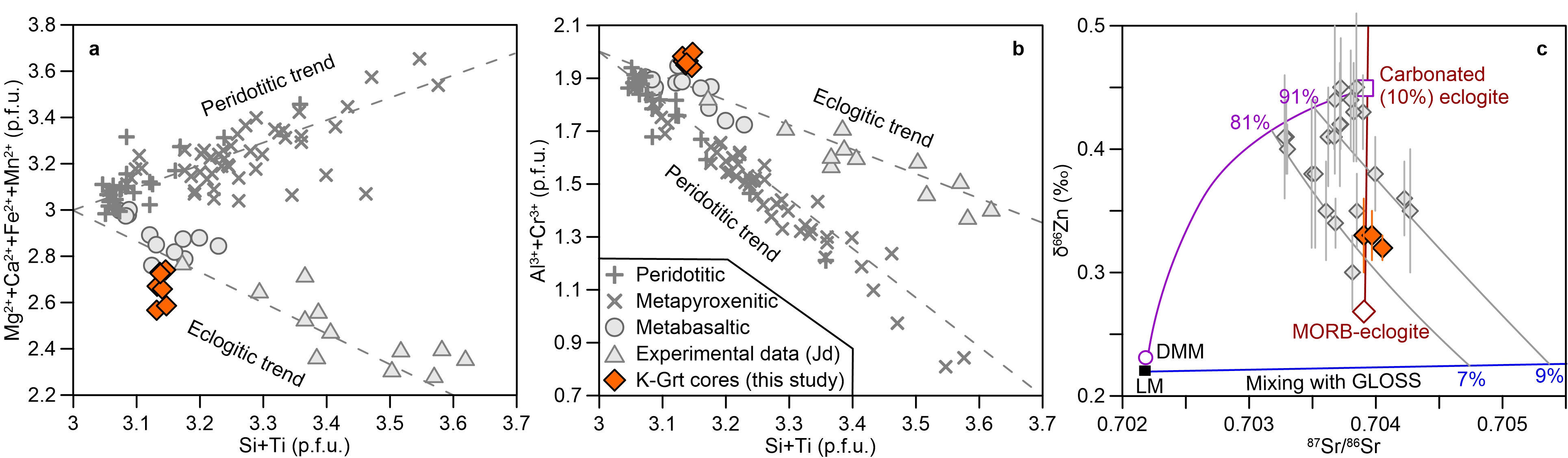


**Supplementary Figure 6. Characteristic compositions and the Zn-Sr isotopes of the decomposed Na-rich majoritic garnets.** (a) Plot of the sum of (Mg^2+^+Ca^2+^+Fe^2+^+Mn^2+^) p.f.u. versus Si + Ti (p.f.u.). (b) Plot of the sum of (Al^3+^+Cr^3+^) p.f.u. versus Si+Ti (p.f.u). Majoritic garnet inclusions in natural diamonds are sourced from Kiseeva et al. (2013)^40^. The experimental data (Jd) are from Bobrov et al. (2008)^23^. All Fe in majoritic garnet is treated as Fe^2+^ for comparison. (c) Zn and Sr isotopic compositions of the decomposed Na-rich majoritic garnets and Cenozoic intraplate basalts from our study area (grey rhombus). The Zn-Sr isotopic compositions of the decomposed garnets are consistent with those of carbonated eclogite. Both the Zn-Sr isotopes of the Cenozoic intraplate basalts and the mixing model are according to Xu et al. (2022)^28^.


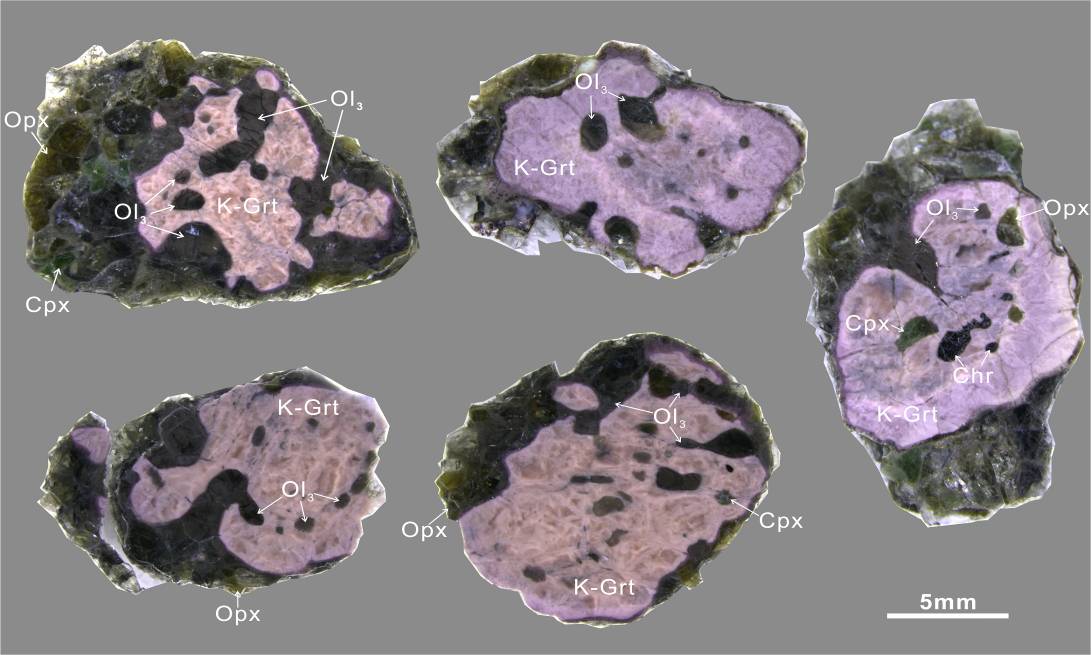


Supplementary Figure 7. Photographs of peridotite fragments depicting the intergrowth of kelyphitized garnet (K-Grt) and olivine (Ol_3_). The olivine grains intergrown with the K-Grt are black, ellipsoidal or irregular in shape. Minor orthopyroxene (Opx), clinopyroxene (Cpx), and chromite (Chr) grains are also present within the intergrowth.


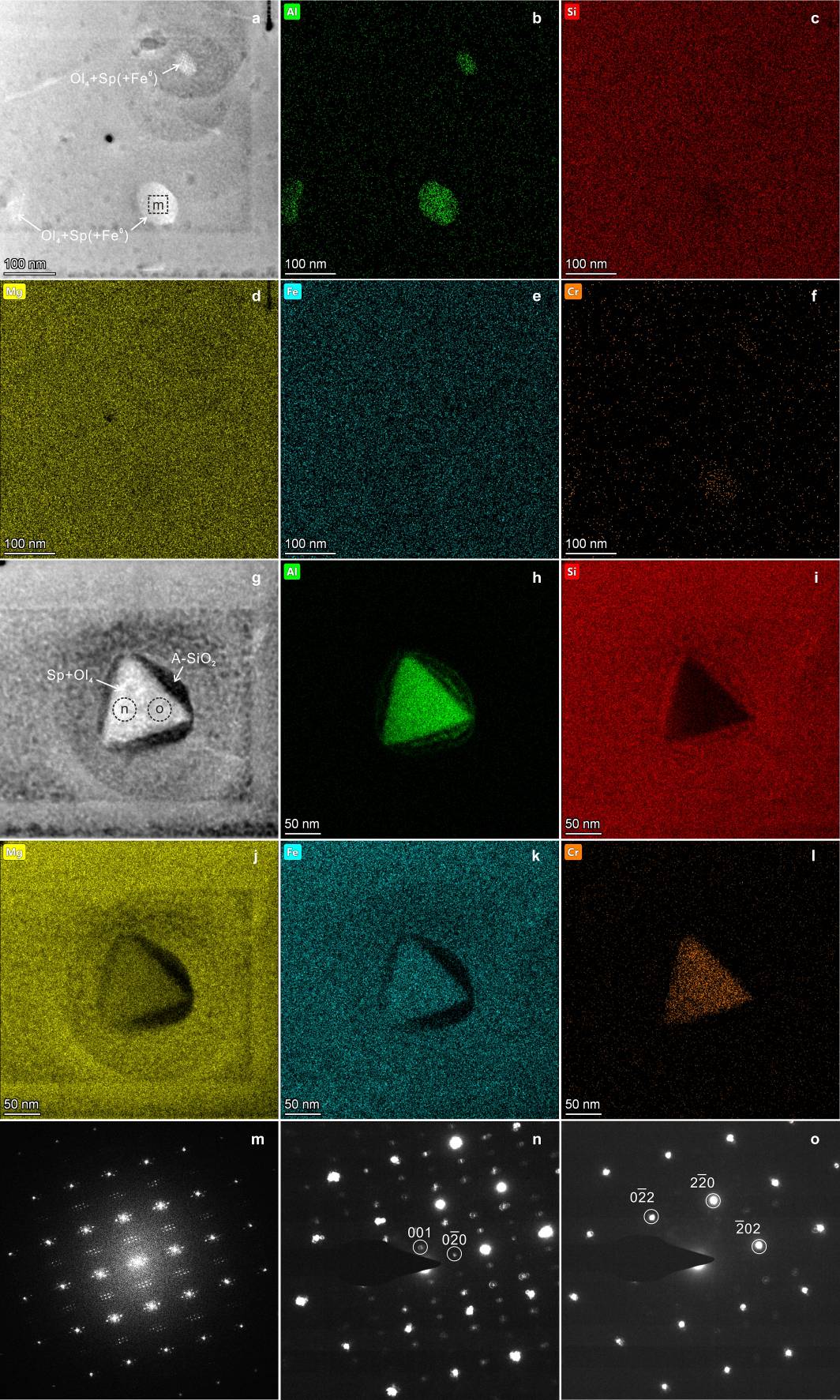


**Supplementary Figure 8.** **Transmission electron microscopy analyses of** **three elliptic inclusions (a-f) and an** **octahedral inclusion (g-l) in olivine-Ol_3_, along with their crystallographic diffraction patterns (m-o).** Panels (a) and (g) are HAADF-STEM micrographs, while panels (b-f) and (h-l) are EDS elemental maps. Panel (m) display a FFT pattern of an elliptic inclusion shown in panel (a). Panels (n) and (o) show NBD patterns of the left and right parts of the octahedra inclusion in panel (g), respectively. The right part of the octahedra inclusion shows a typical spinel diffraction pattern, while the left part of the octahedra inclusion and the examined elliptic inclusion in panel (a) show comparable Mioré diffraction patterns that can be indexed as a mixture of spinel and olivine. Dotted square and circles on panels (a) and (g) are the analytical positions for the FFT and NBD patterns, respectively.

**References**

58 Wessel, P. & Smith, W. H. Free software helps map and display data. Eos, Transactions American Geophysical Union 72, 441-446 (1991).

59 Sun, S. S. & McDonough, W. F. Chemical and isotopic systematics of oceanic basalts: implications for mantle composition and processes. Geological Society, London, Special Publications 42, 313 - 345 (1989).

1. * Corresponding authors. e-mail: wuxiang@cug.edu.cn, jfzhang@cug.edu.cn [↑](#footnote-ref-1)
