# Supplementary material for: Iron disproportionation in peridotite fragments from the mantle transition zone
Source: Nat Commun. 2025 Jul 1;16:5440. doi: 10.1038/s41467-025-60566-y (PMC12215351; doi:10.1038/s41467-025-60566-y)
Supplement: Supplementary file 2 — Description of Additional Supplementary Information [file 41467_2025_60566_MOESM2_ESM.docx]

Supplementary Data for

**Iron disproportionation in peridotite fragments from the mantle transition zone**

Fabin Pan^1^, Xiang Wu^1^[[1]](#footnote-1)^*^, Chao Wang^1^, Yanfei Zhang^1^, Yiping Yang^2^, Xiaobo He^3^, Chong Jin^4^, Lian Zhou^1^, Hongfei Zhang^1^, Hongping He^2^, Junfeng Zhang^1*^

^1^ *State Key Laboratory of Geological Processes and Mineral Resources, and School of Earth Science,* *China University of Geosciences, Wuhan 430074, China*

^2^ *State Key Laboratory of Isotope Geochemistry,* *Guangzhou Institute of Geochemistry, Chinese Academy of Sciences, Guangzhou 510640, China*

^3^ *Department of Ocean Exploration and Technology, Zhejiang Ocean University, Zhoushan 316022, China*

^4^ *Zhejiang Institute of Geosciences, Hangzhou 310007, China*

**Contents of this file**

Supplementary Data 1 to 6

**Introduction**

This file provides the Supplementary Data 1 to 6.

Supplementary Data 1. Major and trace element compositions of the nephelinite host.

Supplementary Data 2. Major element compositions of constituent minerals in the host lherzolites and kelyphitized garnet rims (wt.%)

Supplementary Data 3. Trace element compositions of the kelyphitized garnet cores (ppm)

Supplementary Data 4. Major element compositions of the kelyphitized garnet cores (wt.%)

Supplementary Data 5. Sr, Nd, and Zn isotopic compositions of the kelyphitized garnet cores

Supplementary Data 6. Major element compositions of the olivine (Ol_3_) and its inclusions from STEM-EDS analyses (wt.%)

1. * Corresponding authors. e-mail: wuxiang@cug.edu.cn, jfzhang@cug.edu.cn [↑](#footnote-ref-1)
